# The lack of association between PADI4_94 or PADI4_104 polymorphisms and RF, ACPA and anti-PAD4 in patients with rheumatoid arthritis

**DOI:** 10.1038/s41598-022-15726-1

**Published:** 2022-07-13

**Authors:** Marek Ciesla, Bogdan Kolarz, Dorota Darmochwal-Kolarz

**Affiliations:** Institute of Medical Sciences, Medical College of Rzeszow University, Warzywna 1a Street, 35-310 Rzeszow, Poland

**Keywords:** Rheumatology, Rheumatic diseases, Rheumatoid arthritis, Genetic markers

## Abstract

Rheumatoid arthritis (RA) is a chronic autoimmune disease that leads to chronic inflammation of synovial tissue, ultimately causing joint damage, disability, and premature mortality. The peptidylarginine deiminase (PAD) family of proteins is involved in the production of anticitrullinated peptide antibodies (ACPA), which are clinically relevant markers of RA. ACPA recognizes citrullinated proteins generated mainly by PAD4. Polymorphisms of the *PADI4* gene have been associated with RA in Asian populations, but in Europeans these associations are still difficult to estimate. A total of 147 subjects, 122 patients with RA, 52 ± 12.3 aged, 84.4% women and 25 healthy controls, 53 ± 8.4 aged, 72% women were enrolled in the study. Two single nucleotide polymorphisms (SNPs) of the *PADI4* gene (*PADI4_94*, rs2240340 and *PADI4_104*, rs1748033) were genotyped using a real-time polymerase chain reaction. Genetic models (co-dominant-1 and 2, dominant, over-dominant, and recessive) were applied to find the associations between genotypes and ACPA as well as PAD4 antibodies (anti-PAD4) levels. We found no relationship between the distribution of genotypes in different genetic models and the levels of anti-PAD4, ACPA and RF antibodies. There were also no differences with respect to the haplotypes. Genetic variants *PADI4_94* and *PADI4_104* may not be clinically relevant as prognostic factors in patients with established RA.

## Introduction

Rheumatoid arthritis (RA) is a systemic autoimmune disease that affects about 0.5–1% of the global population^1^. According to American College of Rheumatology/European League Against Rheumatism criteria, the disease is defined as inflammatory arthritis^2^. The pathogenesis of the disease is still unknown; however, it is considered a multistage process that linked genetic factors (e.g. HLA-DRB1 locus), environmental factors (e.g. bacterial and viral infections) and behavioral factors (e.g. smoking, physical activity)^3–6^.

The Peptidyl Arginine Deiminase 4 (*PADI4)* gene is located on chromosome 1 at location 17.308.195–17.364004, on the forward strand with reference to the assembly GRCh38. The gene encodes 5 splice variants and is associated with the development or function of the immune system, chromatin organization, and protein modification processes such as histone alternation (Ensembl database, release 103; ^7^). According to The Human Protein Atlas^8^ (available from: http://www.proteinatlas.org; release date: 2021.02.24) and Ensembl genome browser (version: 92.38), the gene is expressed primarily in the spleen, bone marrow, granulocytes, monocytes, and to a lesser extent is transcripted in T and B cells and dendritic cells. The *PADI4* gene is associated with the formation of neutrophil extracellular traps (NETs) formation^9,^^10^. Previous reports have shown that NETs are related to a variety of autoimmune diseases, including RA. In this process, *PADI4* is responsible for histone modification and promotes chromatin decondensation. Neutrophils secrete NETs that contain extracellular chromatin with histones and granular proteins. This mechanism leads to an antimicrobial effect, but may also be pathological due to the nonspecific nature of NETs that leads to uncontrolled pro-inflammation^11^. The *PADI4* gene is a member of the family genes that encodes the PAD4 enzyme, which is responsible for the post-translational protein citrullination by converting arginine residues to citrulline. Antibodies against citrullinated proteins (ACPA) together with rheumatoid factor (RF) are important markers of RA. Furthermore, PAD4 is the target of anti-PAD4 antibodies (anti-PAD4), that are associated with a more unfavorable course of the disease^12^.

Single nucleotide polymorphisms (SNPs) have been related to susceptibility to RA ^3^. The first and most significant genetic locus associated with the development of the disease are class II human leukocyte antigen (HLA) genes and other non-HLA genes, including: *PADI4*, *IL23R*, *PTPN22* and others ^13^. Previous studies have shown an association between SNPs in *PADI4 gene* and susceptibility to RA mainly in Asian population, but the results are inconsistent amongst Caucasians ^14^.

An SNP with rs2240340 G > A, *PADI4_94,* is located in the intronic region of the *PADI4* gene with a minor allele frequency in the European population (HapMap CEU) of 0.43 ^15^. The association between *PADI4_94* and susceptibility to RA in the Japanese population was reported with an odds ratio (OR) of 1.22 ^16^. On the contrary, this association has not been confirmed with respect to the European population ^17–19^. A SNP with rs1748033 T > C (A > G), *PADI4_104*, is located in the 5' untranslated region with a minor allele frequency in the European population (HapMap CEU) of 0.36. The nucleotide change T > C is responsible for the synonymous leucine change at codon 117 ^15^. Some studies have shown the association between the aforementioned SNP and susceptibility to RA. In the Japanese population, the higher risk of RA development was related especially to male smokers ^20^. No relationship has been found between *PADI4_104* and RA in Caucasians ^19,21,22^. It may appear that both SNPs are linked to the development of RA in Asian population, and there is no association amongst Caucasians, but the meta-analysis conducted by Lee et al. ^14^ has shown a new point of view on the above relation. The *PADI4_94* and *PADI4_104* variants were associated with susceptibility to RA in both populations after implementing a specific genetic model. The aim of this study was to investigate the association between the two polymorphisms *PADI4_94* (rs2240340) and *PADI4_104* (rs1748033) and the levels of anti-PAD4 antibodies in patients with RA, taking into account the different genetic models.

## Results

The prevalence of the *PADI4_94* and *PADI4_104* minor allele (for a total of 122 RA patients) was 0.48: GG = 36 (29.5%), GA = 56 (45.9%), AA = 30 (24.6%) and 0.36: GG = 49 (40.2%), GA = 58 (47.5%), AA = 15 (12.3%), respectively. The distribution of genotypes in patients with RA was consistent with the Hardy–Weinberg equilibrium (*p* = 0.38 and 0.73, respectively). Regarding *PADI4_94*, in the anti-PAD4 positive RA group (n = 68), the prevalence of genotypes was as follows: GG = 17 (25%), GA = 33 (48.5%), AA = 18 (26.5%). In the anti-PAD4 negative group (n = 54) there were 19 homozygotes GG (35.2%), 23 heterozygotes GA (42.6%), and 12 homozygotes AA (22.2%). The distribution of *PADI4_104* genotypes in anti-PAD4 positive patients with RA was as follows: GG = 28 (41.2%, GA = 33 (48.5%) and AA = 7 (10.3%). In the anti-PAD4 negative group there were 21 (38.9%) carriers of GG genotype, 25 (46.3%) carriers of GA and 8 carriers of AA (14.8%). Genotype distribution for the following groups: ACPA positive vs. ACPA negative, and RF positive vs. RF negative can be found in Supplementary Table S1.

In relation to *PADI4_94* in the co-dominant 1 model there was no difference in the level of anti-PAD4 antibodies (*p* = 0.52) between carriers of the GG genotype—median: 558.17 U/ml [interquartile range: 368.94–1002.15] and carriers of the GA genotype 699.74 [368.68–1372.55].

The co-dominant 2 model (GG vs. AA genotypes) also showed no differences: 558.17 [368.94–1002.15] vs. 925.55 [278.16–1815.44], *p* = 0.54. The dominant model (GA + AA vs. GG) showed a lack of association (*p* = 0.47) between anti-PAD4 level and genotype distribution—727.81 [339.36–1398.8] vs. 558.17 [368.94–1002.15], respectively. There was no relationship to the over-dominant model (GA vs. GG + AA, *p* = 0.79): 699.74 [368.68–1372.55] vs. 679.16 [337.02–1286.2]. The recessive model (AA vs. GA + GG) also did not show an association (*p* = 0.66) between anti-PAD4 levels and genotype distribution—925.55 [278.16–1815.44] vs. 677.9 [368.68–1259.8], accordingly.

In relation to *PADI4_104* in the co-dominant-1 model (GG vs. GA), the subgroups had similar levels of anti-PAD4 antibodies: 706.58 [412.17–1286.2] vs. 681.39 [336.22–1233.4]; *p* = 0.82.

In the co-dominant-2 model (genotypes GG vs. AA) there was no difference in the levels of anti-PAD4—706.58 [412.17–1286.2] vs. 564.96 [271.26–1910]; p = 0.85.

Dominant and over-dominant models (genotypes GA + AA vs. GG and GA vs. GG + AA, respectively) also showed an insignificant association between the groups: 680.86 [325.98–1288.1] vs. 706.58 [412.17–1286.2]; *p* = 0.8 and 681.39 [336.22–1233.4] vs. 691.49 [405.61–1372.55]; p = 0.86, accordingly. The recessive model (AA vs. GA + GG) did not show differences in anti-PAD4 levels–564.96 [271.26–1910] vs. 681.92 [346.2–1286.2]; *p* = 0.93. Furthermore, genetic models did not show differences in the levels of other antibodies (ACPA and RF) between seropositive and seronegative patients with RA. For details, please refer to Supplementary Tables S2-S8. Haplotype analysis was performed using the LDhap tool ^23^ (available from https://ldlink.nci.nih.gov/?tab=ldhap). Between *PADI4_94* and *PADI4_104,* 3 functional haplotypes were estimated in the European population: G_G, A_A, and A_G. In our study, the A_G haplotype was represented by 3 cases, therefore it was omitted in further analysis. There were no associations between anti-PAD4, ACPA and RF levels and haplotypes (*p* = 0.99, *p* = 0.36 and *p* = 0.6, respectively).

## Discussion

The present study shows the lack of association between anti-PAD4 antibody levels and the prevalence of genotypes with respect to different genetic models (codominant, dominant, overdominant, and recessive) for both single nucleotide polymorphisms: *PADI4_94* (rs2240340) and *PADI4_104* (rs1748033).

A total of five PAD enzyme isotypes were found, in which PAD2, PAD3, and PAD4 were responsible for autoimmune reactions in RA. PAD4 is the most characterized. It is found mainly in the nucleus of white blood cells, and its overexpression has been found specifically in neutrophils and monocytes in synovial tissue. SNPs in the *PADI4* gene have also been linked to the development of RA in some populations, especially Asian, but amongst Europeans the results are inconclusive ^24,25^. The new light on this issue was presented in the study conducted by Lee et al. ^14^. They showed that the variants *PADI4_94* and *PADI4_104* may be associated with susceptibility to RA in Asian and Caucasian populations when homozygous contrast was used. Consequently, we assumed that the use of genetic models may be helpful in determining the relationship between SNPs and antibody levels. In addition to genetic factors associated with RA, epigenetic changes have been reported in the *PADI4* gene. Increased methylation in the promoter region was associated with lower disease activity, lower levels of ACPA, and anti-PAD4 antibodies ^26^.

Anti-PAD4 antibodies are associated with structural damage of joints and a more severe clinical outcome; therefore, their evaluation may be of prognostic importance ^25^. The specificity of them was reported to be greater than 95% in patients with RA. Antibodies are not specific to RA, as they have been found in other rheumatic diseases, e.g. SLE, but at lower levels ^27^. Anti-PAD4 antibodies have an incidence ranging from 16.2 to 50% of RA patients and may be related to the study population and duration of the disease ^12,24,28–30^. In this study, anti-PAD4 positivity was approximately 56% and may be associated with the duration of the disease (the median duration of the disease was 10 years) and the relatively small size of the group derived from one medical center. Moreover, Reyes-Castillo et al. ^24^ showed that patients with RA, with the disease lasting more than 2 years (mean duration of the disease was 8 years), had higher levels of anti-PAD4 antibodies compared to patients with disease duration of less than 2 years.

We found no association between anti-PAD4 antibody levels and genotypes, which is consistent with recent reports ^12,24^. In the study conducted by Reyes-Castillo et al., three SNPs were tested: *PADI4_89* G > A (rs11203366), *PADI4_90 T* > *C* (rs11203367) and *PADI4_92 G* > *C* (rs874881). They found no association between the susceptibility haplotype GTG and the levels of anti-PAD4 antibodies; however, carriers of the susceptibility haplotype demonstrated higher ACPA levels. On the other hand, a study conducted by Harris et al. ^31^ demonstrated an association between a susceptible haplotype and anti-PAD4 levels with an odds ratio (OR) of 2.59. They genotyped the same three SNPs as Reyes-Castillo et al. Furthermore, when diplotype analysis was applied, carriers of heterozygous genotypes, including both nonsusceptible and susceptible haplotypes, had increased antibody levels compared to patients homozygous in haplotype 1, with an OR of 4.02. It must be emphasized that the mean duration of the disease in RA patients (overall) was longer than in the study conducted by Reyes-Castillo et al. and was 12.5 years. In addition, a study by Guderud et al. ^12^ was focused on the relationship between autoantibody level and genetic factors. They found no association between *PADI4* rs2240340 and rs1635579 and anti-PAD4 autoantibodies. On the other hand, Guderud et al. ^12^ demonstrated that ACPA- negative patients vs. healthy controls showed a weak association with RA morbidity in relation to the two *PADI4* SNPs: rs2240340 (*PADI4_94*) and rs1635579. Both polymorphisms mentioned turned out to be associated with double negative patients with RA (ACPA negative and anti-PAD4 negative). The authors suggest that genetic risk factors should only be evaluated in relation to ACPA status, as also suggested in a previous study ^24^. In this study, we did not confirm the association between SNPs and ACPA, anti-PAD4 and RF antibody levels. Current diagnostic criteria include serological markers, such as RF and ACPA ^2^. ACPA shows positivity in the range of 60–80% of RA with a specificity in the range 90–95% ^32^. In our cohort, less than 15% of patients with RA were ACPA negative. As previously reported, ACPA has been associated with a higher risk of developing RA in healthy individuals and may occur before the clinical symptoms of RA. ACPA-positive RA patients have a less favorable prognosis, including more complicated structural damage and a worse response to therapy. In the present study, no relationship was found between ACPA concentration and genotypes, also in different genetic models.

We did not confirm the hypothesis that carriers of the genetic variants may have a different (higher) autoantibody status and therefore a less favorable clinical outcome, as previously suggested. The limitation of the study was the small size of the group, notably in the context of genotyping. Further investigations in a larger cohort of patients are necessary to confirm our data, especially the conclusions regarding ACPA-negative patients should be confirmed in the wider population. We believe that in relation to established RA, genetic testing, especially including common SNPs, may not be helpful. It seems to be helpful to measure and compare the anti-PAD4 levels with genotypes during the RA diagnosis or in pre-RA.

## Methods

A total of 147 subjects, 122 patients with RA, 52 ± 12.3 aged, 84.4% women and 25 healthy controls, 53 ± 8.4 aged, 72% women were enrolled in the study. Written informed consent was obtained from every participant before entering the study and all research was performed in accordance with relevant guidelines. The study was conducted in accordance with the Declaration of Helsinki and the Ethics Committee of the Medical University of Lublin approved the study (protocol number KE-0254/7/2016). The characteristics of the individuals are presented in Table 1.

**Table 1:** Characteristics of the subjects.

Characteristics	RA overall n = 122	HC n = 25	p-value
Age (years)	52 ± 12.3	53 ± 8.4	0.9
Females; n (%)	103 (84.4)	18 (72)	0.23
Disease duration (years)	10 [3–16]	n/a	n/a
RF-positive; n (%),	82 (67.2)	none	n/a
ACPA-positive; n (%)	104 (85.2)	none	n/a
anti-PAD4-positive; n (%)	68 (55.7)	2 (8)	** < 0.0001**
ESR [mm/h]	26 [8–57.5]	15.5 [7–20]	**0.001**
CRP [mg/dl]	8.54 [0.53–19.12]	0.59 [0.2–1.95]	** < 0.0001**
DAS28	3.95 ± 1.55	n/a	n/a
Swollen joints	1 [0–4]	n/a	n/a
Tender joints	3 [1–6]	n/a	n/a
VAS PGA	27 [9–61]	n/a	n/a
VAS PhGA	20 [7–49]	n/a	n/a

Data are presented as mean ± SD; median [interquartile range] or number (%). Abbreviations: ACPA, anti-citrullinated protein antibodies; CRP, C-reactive protein; PAD4, peptidyl arginine deiminase type 4; ESR, erythrocyte sedimentation rate; HC, healthy controls; RA, patients with rheumatoid arthritis; RF, rheumatoid factor; VAS PhGA, visual analog scale physician global assessment; VAS PGA, visual analog scale patient global assessment.

Significant values are in [bold].

Whole blood was collected and stored at -80 °C until analysis. DNA was extracted from 200 µl of sample according to the manufacturer’s protocol using the GeneMATRIX Quick Blood DNA Purification Kit (Eurx, Poland).

Genotypes of *PADI4_94* with rs2240340 (assay ID: C__16176717_10, Thermo Fisher Scientific, USA) and *PADI4_104* with rs1748033 (assay ID: C___7541083_1, Thermo Fisher Scientific, USA) were evaluated using TaqMan Genotyping assays and the Endpoint Genotyping module of the COBAS z480 Real-Time PCR System (LightCycler 480 SW, version 1.5.1.62 SP2–UDF v.2.0.0, Roche, Germany). Antibodies levels were previously evaluated and the methodology was described ^33^. Briefly, ACPA and RF cut-offs were estimated according to manufacturer’s recommendations. Anti-PAD4 positivity was evaluated based on results in a control group as below 95^th^ percentile. Additionally for PAD4 a receiver operating characteristic (ROC) curve with cut-off value (Supplementary Figure S1) was performed to confirm previously determined threshold. Both cut-offs are slightly different (615.24 vs. 628.5 U/mL), however it does not affect the results.

Quantitative values were presented as mean ± standard deviation (SD) or median [interquartile range]. Differences between two independent groups were compared using the Student's t test or the Mann–Whitney U test. Differences between more than two groups were evaluated using the Kruskal–Wallis ANOVA test. The qualitative parameters are given as numbers with percentages and were evaluated using the contingency tables with the χ^2^ test with Yates’s correction. A p-value of less than 0.05 was considered statistically significant. Logistic regression was used to estimate the odds ratio (OR) and confidence interval (CI). The analysis was performed with STATISTICA version 13 (Dell Inc. 2016). The ROC curve analysis was performed by MedCalc version 20.111 (MedCalc Software Ltd, Ostend, Belgium).

## Supplementary Information

Supplementary Information.

## Data Availability

Data generated and analysed during the current study are available in the manuscript and in the supplementary material.
