# Supplementary material for: The lack of association between PADI4_94 or PADI4_104 polymorphisms and RF, ACPA and anti-PAD4 in patients with rheumatoid arthritis
Source: Sci Rep. 2022 Jul 13;12:11882. doi: 10.1038/s41598-022-15726-1 (PMC9279283; doi:10.1038/s41598-022-15726-1)
Supplement: Supplementary file 1 — Supplementary Information. [file 41598_2022_15726_MOESM1_ESM.docx]

# **Supplementary data**

**The lack of association between PADI4_94 or PADI4_104 polymorphisms and RF, ACPA and anti-PAD4 in patients with rheumatoid arthritis.**

| ***PADI4* SNPs** |  | Number of carriers; % | | | | | | | |
| --- | --- | --- | --- | --- | --- | --- | --- | --- | --- |
| ***PADI4_94***  **rs2240340** | **Allele/**  **Genotype** | **RA ACPA-positive**  **n=104** | **RA ACPA-negative**  **n=18** | **RA**  **anti-PAD4-positive**  **n=68** | **RA**  **anti-PAD4-negative**  **n=54** | **RA RF-positive**  **n=82** | **RA RF-negative**  **n=40** | **RA overall,**  **n = 122** | **Controls,**  **n = 25** |
|  | **MAF (A allele)** | 48.6% | 41.7% | 50.7% | 43.5% | 48.2% | 46.3% | 47.5% | 44% |
|  | GG | 30; 28.8% | 6; 33.3% | 17; 25% | 19; 35.2% | 24; 29.3% | 12; 30% | 36; 29.5% | 6; 24% |
|  | GA | 47; 45.2% | 9; 50% | 33; 48.5% | 23; 42.6% | 37; 45.1% | 19; 47.5% | 56; 45.9% | 16; 64% |
|  | AA | 27; 26% | 3; 16.7% | 18; 26.5% | 12; 22.2% | 21; 25.6% | 9; 22.5% | 30; 24.6% | 3; 12% |
| ***PADI4_104***  **rs1748033** | **Allele/Genotype** |  | | | | | | | |
|  | **MAF (A allele)** | 35.1% | 41.7% | 34.6% | 38% | 35.4% | 37.5% | 36.1% | 26% |
|  | GG | 43; 41.3% | 6; 33.3% | 28; 41.2% | 21; 38.9% | 33; 40.2% | 16; 40% | 49; 40.2% | 13; 52% |
|  | GA | 49; 47.1% | 9; 50% | 33; 48.5% | 25; 46.3% | 40; 48.8% | 18; 45% | 58; 47.5% | 11; 44% |
|  | AA | 12; 11.5% | 3; 16.7% | 7; 10.3% | 8; 14.8% | 9; 11% | 6; 15% | 15; 12.3% | 1; 4% |

Table S1. Distribution of genotypes and alleles in PADI4_94 rs2240340 and PADI_104 rs1748033 in the studied groups.

Data are presented as number; %. Abbreviations: ACPA, anti-citrullinated protein antibodies; anti-PAD4, anti-peptidyl arginine deiminase type 4 antibodies; MAF, minor allele frequency; RA, patients with rheumatoid arthritis; RF, rheumatoid factor; SNP, single nucleotide polymorphism.

Table S2. Distribution of genotypes in PADI4_94 rs2240340 and related antibodies levels in a different genetic models between ACPA-positive and ACPA-negative patients with rheumatoid arthritis.

| ***PADI4_94***  rs2240340 | **RA ACPA-positive**  **n=104** | **RA ACPA-negative**  **n=18** | **Chi^2^ with Yate’s correction**  **P-value** | **OR [95% CI]** | **P-value** | **ACPA level [U/ml]** | **P-value** |
| --- | --- | --- | --- | --- | --- | --- | --- |
| **Co-dominant 1** GG vs.GA | 30; 28.8%  **vs**.  47; 45.2% | 6; 33.3%  **vs.**  9; 50% | 0.83 | 0.96 [0.31 – 3] | 0.94 | 431.55 [135.94 – 991.95]  **vs.**  468.1 [102.69 – 1097.55] | 0.66 |
| **Co-dominant 2** GG vs. AA | 30; 28.8%  **vs.**  27; 26% | 6; 33.3%  **vs.**  3; 16.7% | 0.67 | 0.56 [0.12 – 2.51] | 0.43 | 431.55 [135.94 – 991.95]  **vs.**  265.87 [100.09 – 913.44] | 0.62 |
| **Dominant** GA+AA vs. GG | 74; 71.2%  **vs.**  30; 28.8% | 12; 66.7%  **vs.**  6; 33.3% | 0.92 | 1.23 [0.42 – 3.63] | 0.7 | 442.81 [102.61 – 1025.1]  **vs.**  431.55 [135.94 – 991.95] | 0.93 |
| **Over-dominant** GA vs. GG+AA | 47; 45.2%  **vs.**  57; 54.8% | 9; 50%  **vs.**  9; 50% | 0.9 | 0.82 [0.3 – 2.27] | 0.71 | 468.1 [102.69 – 1097.55]  **vs.**  403.58 [104.51 – 913.8] | 0.51 |
| **Recessive**  AA vs. GA+GG | 27; 26%  **vs.**  77; 74% | 3; 16.7%  **vs.**  15; 83.3% | 0.58 | 0.57 [0.15 – 2.15] | 0.38 | 265.87 [100.09 – 913.44]  **vs.**  442.9 [106.68 – 1028.5] | 0.5 |

Data are presented as number; % or median [interquartile range]. Abbreviations: CI, confidence interval; OR, odds ratio; for other please refer to table S1.

Table S3. Distribution of genotypes in PADI4 rs1748033 and related antibodies levels in a different genetic models between ACPA-positive and ACPA-negative patients with rheumatoid arthritis.

| ***PADI4_104***  rs1748033 | **RA ACPA-positive**  **n=104** | **RA ACPA-negative**  **n=18** | **Chi^2^ with Yate’s correction**  **P-value** | **OR [95% CI]** | **P-value** | **ACPA level [U/ml]** | **P-value** |
| --- | --- | --- | --- | --- | --- | --- | --- |
| **Co-dominant 1** GG vs.GA | 43; 41.3%  **vs.**  49; 47.1% | 6; 33.3%  **vs.**  9; 50% | 0.84 | 1.32 [0.43 – 4.05] | 0.63 | 443.37 [149.05 – 957.99]  **vs.**  444.39 [102.61 – 1143.1] | 0.96 |
| **Co-dominant 2** GG vs. AA | 43; 41.3%  **vs**.  12; 11.5% | 6; 33.3%  **vs.**  3; 16.7% | 0.74 | 1.79 [0.38 – 8.5] | 0.46 | 443.37 [149.05 – 957.99]  **vs.**  199.98 [49.08 – 913.44] | 0.31 |
| **Dominant** GA+AA vs. GG | 61; 58.7%  **vs.**  43; 41.3% | 12; 66.7%  **vs.**  6; 33.3% | 0.7 | 0.71 [0.24 – 2.06] | 0.52 | 403.19 [100.09 – 1025.1]  **vs.**  443.37 [149.05 – 957.99] | 0.71 |
| **Over-dominant** GA vs. GG+AA | 49; 47.1%  **vs.**  55; 52.9% | 9; 50%  **vs.**  9; 50% | 0.98 | 1.12 [0.41 – 3.08] | 0.82 | 444.39 [102.61 – 1143.1**]**  **vs.**  403.58 [113.67 – 913.62] | 0.74 |
| **Recessive**  AA vs. GA+GG | 12; 11.5%  **vs.**  92; 88.5% | 3; 16.7%  **vs.**  15; 83.3% | 0.82 | 1.53 [0.38 – 6.17] | 0.56 | 199.98 [49.08 – 913.44]  **vs.**  443.37 [110.6 – 1031.1] | 0.28 |

Data are presented as number; % or median [interquartile range]. Abbreviations: please refer to tables S1 and S2.

Table S4. Distribution of genotypes in rs2240340 and related antibodies levels in a different genetic models between anti-PAD4-positive and anti-PAD4-negative patients with rheumatoid arthritis.

| ***PADI4_94***  rs2240340 | **RA**  **anti-PAD4-positive**  **n=68** | **RA**  **anti-PAD4-negative**  **n=54** | **Chi^2^ with Yate’s correction**  **P-value** | **OR [95% CI]** | **P-value** | **anti-PAD4 level [U/ml]** | **P-value** |
| --- | --- | --- | --- | --- | --- | --- | --- |
| **Co-dominant 1** GG vs.GA | 17; 25%  **vs.**  33; 48.5% | 19; 35.2%  **vs.**  23; 42.6% | 0.38 | 0.62 [0.27- 1.47] | 0.27 | 558.17 [368.94 – 1002.15]  **vs.**  699.74 [368.68 – 1372.55 ] | 0.52 |
| **Co-dominant 2** GG vs. AA | 17; 25%  **vs.**  18; 26.5% | 19; 35.2%  **vs.**  12; 22.2% | 0.43 | 0.59 [0.22-1.62] | 0.3 | 558.17 [368.94 - 1002.15]  **vs.**  925.55 [278.16 – 1815.44] | 0.54 |
| **Dominant** GA+AA vs. GG | 51; 75%  **vs**.  17; 25% | 35; 64.8%  **vs.**  19; 35.2% | 0.31 | 0.61 [0.28-1.35] | 0.22 | 727.81 [339.36 – 1398.8]  **vs.**  558.17 [368.94 – 1002.15] | 0.47 |
| **Over-dominant** GA vs. GG+AA | 33; 48.5%  **vs.**  35; 51.5% | 23; 42.6%  **vs.**  31; 57.4% | 0.64 | 0.79 [0.38-1.63] | 0.51 | 699.74 [368.68 – 1372.55 ]  **vs.**  679.16 [337.02 – 1286.2] | 0.79 |
| **Recessive**  AA vs. GA+GG | 18; 26.5%  **vs.**  50; 73.5% | 12; 22.2%  **vs.**  42; 77.8% | 0.74 | 0.79 [0.34-1.85] | 0.59 | 925.55 [278.16 – 1815.44]  **vs.**  677.9 [368.68 – 1259.8] | 0.66 |

Data are presented as number; % or median [interquartile range]. Abbreviations: please refer to tables S1 and S2.

Table S5. Distribution of genotypes in rs1748033 and related antibodies levels in a different genetic models between anti-PAD4-positive and anti-PAD4-negative patients with rheumatoid arthritis.

| ***PADI4_104***  rs1748033 | **RA**  **anti-PAD4-positive**  **n=68** | **RA**  **anti-PAD4-negative**  **n=54** | **Chi^2^ with Yate’s correction**  **P-value** | **OR [95% CI]** | **P-value** | **anti-PAD4 level [U/ml]** | **P-value** |
| --- | --- | --- | --- | --- | --- | --- | --- |
| **Co-dominant 1** GG vs.GA | 28; 41.2%  **vs.**  33; 48.5% | 21; 38.9%  **vs.**  25; 46.3% | 0.86 | 0.99 [0.45 – 2.19] | 0.98 | 706.58 [412.17 – 1286.2]  **vs.**  681.39 [336.22 – 1233.4] | 0.82 |
| **Co-dominant 2** GG vs. AA | 28; 41.2%  **vs.**  7; 10.3% | 21; 38.0%  **vs.**  8; 14.8% | 0.68 | 1.52 [0.47 – 4.98] | 0.48 | 706.58 [412.17 – 1286.2]  **vs.**  564.96 [271.26 – 1910] | 0.85 |
| **Dominant** GA+AA vs. GG | 40; 58.8%  **vs.**  28; 41.2% | 33; 61.1%  **vs.**  21; 38.9% | 0.94 | 0.91 [0.43 – 1.9] | 0.8 | 680.86 [325.98 – 1288.1]  **vs.**  706.58 [412.17 – 1286.2] | 0.8 |
| **Over-dominant** GA vs. GG+AA | 33; 48.5%  **vs.**  35; 51.5% | 25; 46.3%  **vs.**  29; 53.7% | 0.95 | 0.91 [0.44 – 1.88] | 0.81 | 681.39 [336.22 – 1233.4]  **vs.**  691.49 [405.61 – 1372.55] | 0.86 |
| **Recessive**  AA vs. GA+GG | 7;10.3%  **vs.**  61;89.7% | 8; 14.8%  **vs.**  46; 85.2% | 0.63 | 1.52 [0.51 – 4.53] | 0.45 | 564.96 [271.26 – 1910]  **vs.**  681.92 [346.2 – 1286.2] | 0.93 |

Data are presented as number; % or median [interquartile range]. Abbreviations: please refer to tables S1 and S2.

Table S6. Distribution of genotypes in rs2240340 and related antibodies levels in a different genetic models between RF-positive and RF-negative patients with rheumatoid arthritis.

| ***PADI4_94***  rs2240340 | **RA RF-positive**  **n=82** | **RA RF-negative**  **n=40** | **Chi^2^ with Yate’s correction**  **P-value** | **OR [95% CI]** | **P-value** | **RF level [U/ml]** | **P-value** |
| --- | --- | --- | --- | --- | --- | --- | --- |
| **Co-dominant 1** GG vs.GA | 24; 29.3%  **vs.**  37; 45.1% | 12; 30%  **vs.**  19; 47.5% | 0.87 | 1.03 [0.42 – 2.53] | 0.96 | 39.23 [13.79 – 70.71]  **vs.**  47.73 [16.68 – 83.06] | 0.87 |
| **Co-dominant 2** GG vs. AA | 24; 29.3%  **vs.**  21; 25.6% | 12; 30%  **vs.**  9; 22.5% | 0.98 | 0.86 [0.3 – 2.48] | 0.77 | 39.23 [13.79 – 70.71]  **vs.**  38.07 [15.51 – 61.08] | 0.63 |
| **Dominant** GA+AA vs. GG | 58; 70.7%  **vs.**  24; 29.3% | 28; 70%  **vs.**  12; 30% | 0.9 | 0.97 [0.42 – 2.23] | 0.93 | 41.89 [16.01 – 73.67  **vs.**  39.23 [13.79 – 70.71] | 0.93 |
| **Over-dominant** GA vs. GG+AA | 37; 45.1%  **vs.**  45; 54.9% | 19; 47.5%  **vs.**  21; 52.5% | 0.96 | 1.1 [0.51 – 2.37] | 0.8 | 47.73 [16.68 – 83.06]  **vs.**  38.88 [15.16 – 67.99] | 0.63 |
| **Recessive**  AA vs. GA+GG | 21; 25.6%  **vs.**  61; 74.4% | 9; 22.5%  **vs.**  31; 77.5% | 0.88 | 0.84 [0.34 – 2.08] | 0.71 | 38.07 [15.51 – 61.08]  **vs.**  44.03 [15.67 – 79.15] | 0.52 |

Data are presented as number; % or median [interquartile range]. Abbreviations: please refer to tables S1 and S2.

Table S7. Distribution of genotypes in rs1748033 and related antibodies levels in a different genetic models between RF-positive and RF-negative patients with rheumatoid arthritis.

| ***PADI4_104***  rs1748033 | **RA RF-positive**  **n=82** | **RA RF-negative**  **n=40** | **Chi^2^ with Yate’s correction**  **P-value** | **OR [95% CI]** | **P-value** | **RF level [U/ml]** | **P-value** |
| --- | --- | --- | --- | --- | --- | --- | --- |
| **Co-dominant 1** GG vs.GA | 33; 40.2%  **vs.**  40; 48.8% | 16; 40%  **vs.**  18; 45% | 0.98 | 1.08 [0.47 – 2.46] | 0.86 | 38.72 [15.16 – 68.16]  **vs.**  41.89 [17.35 – 81.37] | 0.68 |
| **Co-dominant 2** GG vs. AA | 33; 40.2%  **vs.**  9; 11% | 16; 40%  **vs.**  6; 15% | 0.83 | 0.73 [0.22 – 2.46] | 0.6 | 38.72 [15.16 – 68.16]  **vs.**  48.68 [8.37 – 64.07] | 0.69 |
| **Dominant** GA+AA vs. GG | 49; 59.8%  **vs.**  33; 40.2% | 24; 60%  **vs.**  16; 40% | 0.86 | 1.01 [0.45 – 2.25] | 0.98 | 43.11 [16.01 – 76.93]  **vs.**  38.72 [15.16 – 68.16] | 0.83 |
| **Over-dominant** GA vs. GG+AA | 40; 48.8%  **vs.**  42; 51.2% | 18; 45%  **vs.**  22; 55% | 0.84 | 0.86 [0.4 – 1.85] | 0.69 | 41.89 [17.35 – 81.37]  **vs.**  39.23 [13.79 – 67.59] | 0.56 |
| **Recessive**  AA vs. GA+GG | 9; 11%  **vs.**  73; 89% | 6; 15%  **vs.**  34; 85% | 0.73 | 1.43 [0.47 – 4.39] | 0.53 | 48.68 [8.37 – 64.07]  **vs.**  39.73 [15.86 – 73.67] | 0.57 |

Data are presented as number; % or median [interquartile range]. Abbreviations: please refer to tables S1 and S2

Table S8. Antibodies levels in regards to the PADI4 genotypes.

| ***PADI4* polymorphism** | **Antibody** | **Genotype** | | | **P-value** |
| --- | --- | --- | --- | --- | --- |
|  |  | **GG, n=36** | **GA, n=56** | **AA, n=30** |  |
| ***PADI4_94***  **rs2240340** | anti-PAD4 [U/ml] | 558.17 [368.94 – 1002.15] | 699.74 [368.68 – 1372.55] | 925.55 [278.16 – 1815.44] | 0.76 |
|  | ACPA [U/ml] | 431.55 [135.94 – 991.95] | 468.1 [102.69 – 1097.55] | 265.87 [100.09 – 913.44] | 0.75 |
|  | RF [U/ml] | 39.23 [13.79 – 70.71] | 47.73 [16.68 – 83.06] | 38.07 [15.51 – 61.08] | 0.8 |
| ***PADI4_104***  **rs1748033** |  | **GG, n=49** | **GA, n=58** | **AA, n=15** | **P-value** |
|  | anti-PAD4 [U/ml] | 706.58 [412.17 – 1286.2] | 681.39 [336.22 – 1233.4] | 564.96 [271.26 – 1910.0] | 0.97 |
|  | ACPA [U/ml] | 443.37 [149.05 – 957.99] | 444.39 [102.61 – 1143.1] | 199.98 [49.08 – 913.44] | 0.56 |
|  | RF [U/ml] | 38.72 [15.16 – 68.16] | 41.89 [17.35 – 81.37] | 48.68 [8.37 – 64.07] | 0.78 |

Data are presented as median [interquartile range]. Abbreviations: please refer to tables S1.

Figure S1. A receiver operating characteristic (ROC) curve for anti-PAD4. The red dot indicates the Youden index.

Abbreviations: AUC, area under the curve; PAD4, peptidyl arginine deiminase type 4.
